# Plant MicroRNAs—Novel Players in Natural Medicine?

**DOI:** 10.3390/ijms18010009

**Published:** 2016-12-22

**Authors:** Anna Lukasik, Piotr Zielenkiewicz

**Affiliations:** 1 Institute of Biochemistry and Biophysics, Polish Academy of Sciences, Pawinskiego 5a, 02-106 Warsaw, Poland; alukasik@ibb.waw.pl; 2 Department of Plant Molecular Biology, Institute of Experimental Plant Biology and Biotechnology, University of Warsaw, Miecznikowa 1, 02-096 Warsaw, Poland

**Keywords:** microRNA (miRNA), plant, cross-kingdom, gene expression regulation, human, natural medicine, diseases

## Abstract

MicroRNAs (miRNAs) represent a class of small non-coding RNAs that act as efficient gene expression regulators and thus play many important roles in living organisms. Due to their involvement in several known human pathological and pathogenic states, miRNA molecules have become an important issue in medicine and gained the attention of scientists from the pharmaceutical industry. In recent few years, a growing number of studies have provided evidence that miRNAs may be transferred from one species to another and regulate gene expression in the recipients’ cells. The most intriguing results revealed that stable miRNAs derived from food plants may enter the mammals’ circulatory system and, after reaching the target, inhibit the production of specific mammalian protein. Part of the scientific community has perceived this as an attractive hypothesis that may provide a foundation for novel therapeutic approaches. In turn, others are convinced about the “false positive” effect of performed experiments from which the mentioned results were achieved. In this article, we review the recent literature that provides evidence (from both fronts) of dietary, plant miRNA uptake and functionality in various consumers. Additionally, we discuss possible miRNA transport mechanisms from plant food sources to human cells.

## 1. Introduction

### 1.1. MicroRNA Function

MicroRNAs (miRNAs) are a class of small (18–24 nt), single-stranded molecules that are evolutionarily conserved among many known species. These non-coding particles mediate posttranscriptional gene expression through target mRNA translation inhibition or degradation by exonuclease action, decapping, or deadenylation of the poly(A) tail [1,2,3,4]. It is estimated that more than 60% of human protein-coding genes contain at least one conserved miRNA binding site and numerous non-conserved sites [5]. Thus, it is not surprising that through their action, miRNAs control crucial biological processes, such as developmental timing, apoptosis, metabolism, immune responses, hormone signaling, cell proliferation, differentiation, and many others [6,7,8,9]. In plants, miRNAs also play roles in flowering, polarity, nutrient homeostasis, phase change, biotic and abiotic stress responses [10,11].

Using the rapidly advancing experimental and computational methods, especially high-throughput sequencing approaches, miRNA molecules have been found in a wide spectrum of organisms, ranging from viruses to algae, plants and animals [12,13,14,15]. Recent reports have shown that miRNA-like molecules are also present in some fungi [16,17]. Overall, to date more than 35,000 mature miRNAs from 223 species have been annotated in the miRBase database (release 21) [18]. Growing interest in miRNAs and technical capabilities have enabled researchers to identify these intriguing molecules not only in various tissues, cells and organs but also in different body fluids, e.g., serum [19,20,21], plasma [19,22,23], urine [24,25,26], breast milk [27,28,29], saliva [30,31], tears [32], semen [33,34], and others [32,35]. Contrary to traditional beliefs on extracellular RNA stability, circulating miRNAs are shown to be resistant to RNase activity and highly stable in extreme pH and temperature conditions [21,36,37,38]. More importantly, it has been reported that the profiles of miRNAs, especially in serum, plasma and urine, are tightly correlated with various diseases and pathological states, including cancer, diabetes, inflammation, infections and tissue injuries [20,24,39,40,41,42,43,44]. Thus, miRNA molecules become new biomarkers to diagnose, prognosis, and monitor patients’ treatment responses [19,45,46,47,48].

Concerning the abundance of miRNAs in biological fluids, a very interesting aspect is miRNA identification in breast milk. Breastfeeding is an essential process in infant health and development, especially immune system development [49,50,51]. Several studies have shown that membranous vesicles, called exosomes, that are present in human milk and colostrum are enriched in miRNAs [27,29,52,53]. miRNA “shipment” in mentioned carriers was proven to protect these molecules from harsh conditions present in the human gastrointestinal (GI) tract. Thus, researchers have assumed that miRNAs may be transferred to the bodies of infants via the GI tract and are likely to contribute to its development as well as protection against infections [27,29,52]. miRNA molecules, namely those that are bovine-derived, have been also identified in commercially available infant formula and various milk products [54,55,56]. The same as human molecules, the mentioned miRNAs from raw bovine milk were shown to be packed in exosomes which protect them from degradation [57,58,59,60]. However, as reported by Howard et al., the pasteurization, homogenization and other processing of milk reduces miRNA concentrations [61]. Recent findings [62] suggest that certain bovine milk-derived miRNAs are bioavailable and appear in adult human blood after consumption. Additionally, one of these miRNAs (miR-29) was shown to regulate specific human gene expression [62]. This report was however questioned [63,64] by other authors.

### 1.2. miRNAs in Therapy—Current Knowledge

Due to their involvement in various biological processes and thus in several human diseases, miRNA molecules quickly became interesting to the pharmaceutical industry. Although drug discovery has often been successful, developing new therapeutics is now becoming an increasingly expensive business, in turn, a large number of disease-related proteins cannot be targeted or modulated by a drug molecule. These “undruggable” proteins are not the only challenge facing the pharmaceutical industry. Despite their confirmed efficacy, the available therapeutics for many known diseases still present significant adverse effects. Therefore, in view of miRNA involvement in the regulation of gene expression, it is not surprising that the attention of scientists from the pharmaceutical industry has shifted to miRNA molecules. Currently, 23 years after the first report of the existence of miRNA [65,66], several miRNA-related drugs are in clinical trials or are even close to reaching the market (e.g., Miravirsen and MRX34) [67,68,69]. These miRNA-based therapeutics comprise mainly two approaches: (1) miRNA inhibition—synthetic single-stranded RNAs (called anti-miRs), which antagonize the action of endogenous miRNA and lead to the upregulation of the specific protein population; and (2) miRNA enhancement—synthetic miRNAs (called miRNA mimics), which are used to mimic endogenous miRNAs and therefore achieve the same function by inhibiting the translation/mediating the degradation of target mRNAs [67,68,70].

Although the previously described approaches may sound easy to introduce, in practice, their development presents many challenges, mainly off-target effects, poor stability and inefficient delivery. To overcome these barriers, several advanced strategies have been investigated and introduced; for example, a variety of RNA chemical modifications can successfully enhance the stability of the molecule and reduce off-target effects. The major types of chemical modifications used in miRNA-related therapies include: (1) phosphorothioate (PS) backbone modification; (2) ribose 2′-OH group modifications (such as the 2′-*O*-methyl group, which is present natively in plant miRNAs); and (3) locked (LNA) or unlocked (UNA) nucleic acids. Combinations of different modification approaches are also very popular [68,71]. Although the mentioned modifications can improve the stability and reduce off-target effects, the effective delivery of therapeutic miRNA molecules is still challenging. Many therapies tested in clinical trials have used viral vectors to deliver RNA molecules, e.g., adenoviruses, adeno-associated viruses and lentiviruses [68,72]. Because there are serious concerns related to this strategy, such as immunogenicity or risk of insertional mutagenesis, the attention of researchers has focused on nonviral vectors. Two recently intensively investigated categories of delivery systems are: (1) lipid-based; and (2) polymer-based vectors, particularly polyethylenimine (PEI)-based delivery systems, dendrimers, and poly(lactide-*co*-glycolide) (PLGA) particles. In addition to synthetic materials, naturally occurring ones, such as chitosan, protamine and atelocollagen, have been used for RNA delivery purposes [68,70,73]. Concerning natural transport vesicles, some labs have shown that self-derived exosomes, as well as exosome-like nanoparticles derived from grapefruit, grape and bovine milk, can serve as ideal cargo for drug delivery, including miRNA-based therapeutics [74,75,76,77]. The delivery method with the use of self-derived or natural exosomes is very appealing and promising; however, at the same time, nontrivial. It was shown that unmodified exosomes administered systematically to the animal organism accumulate in the liver, are rapidly cleared by renal system or deliver their cargo to unintended tissues [78,79]. The efficiency of exosomes targeting specific tissues can be successfully enhanced by displaying homing peptides or ligands on the surface of the exosomes that will target the recipient cell bearing cognate receptor [80,81,82,83]. Various targeting peptides can have different affinity or can be cleaved/degraded, losing their target capability. Therefore, mentioned modifications should be carefully selected to fully perform the desired function [84].

### 1.3. Cross-Kingdom Gene Expression Regulation by miRNAs

Growing interest in miRNA molecules since their discovery in 1993 led to the uncovering of their ability to transfer and regulate gene expression in a cross-kingdom manner, namely, affecting the organism from which they do not originate. To this day, the discussed field has primarily been dominated by viruses, which, through their miRNA molecules, are able to not only enter the latent phase, thus avoiding the host immune response, but also control specific processes in host cells and facilitate the process of infection. There are several well-known examples of animal virus miRNA–host interactions. One of them is the miR-BART5 molecule encoded by the Epstein-Barr virus (EBV), which inhibits the production of the pro-apoptotic p53-upregulated modulator of apoptosis (PUMA) protein and thus enables the infected cells to avoid elimination by apoptosis [85]. Recent studies have provided evidence of the existence of miRNAs that exhibit anti-viral properties. The miR-32 molecule, which is able to block the replication of the primate foamy virus type 1 (PFV-1) in humans, is an example [86]. In turn, the cytoplasmic miRNA cluster, consisting of miR-28, miR-125b, miR-150, miR-223 and miR-382 molecules, interacts with human immunodeficiency 1 virus (HIV-1) in non-activated T CD4+ lymphocytes and inhibits its multiplication [87].

Interesting work concerning this topic was conducted by LaMonte et al., who showed that two human miRNAs (miR-451 and let-7i) highly enriched in erythrocytes carrying a variant hemoglobin allele (HbS) are able to translocate into the malaria parasite *Plasmodium falciparum*. Moreover, these miRNAs can fuse to the parasite’s mRNA, inhibit its translation and eventually affect *P. falciparum*’s biology and survival [88]. Because erythrocytes carrying the mentioned variant hemoglobin allele cause sickle cell disease and are resistant to malaria infection, LaMonte et al. suggested that investigated miRNA’s activity may represent a novel host defense strategy against this pathogen [88].

A different level of cross-kingdom gene expression regulation by miRNAs was presented by Zhang et al. in an intriguing study in 2012. In their publication, the authors provide evidence that the plant miRNA MIR168a from a food source, namely *Oryza sativa* (rice), is present and stable in human serum [89]. Additionally, they showed that MIR168a targets the mRNA of the low-density lipoprotein receptor adaptor protein 1 (LDLRAP1). This miRNA was able to reduce the LDLRAP1 protein level in the blood and liver of mice fed rice, which eventually resulted in an increase in low-density lipoproteins (LDL) in their plasma [89]. Zhang et al. revealed also that more than half of the MIR168a in serum is abundant in microvesicles (MV) and that the mentioned molecules can use the mammalian Argonaute 2 (AGO2) protein to form the RNA-induced silencing complex (RISC) and execute their functions. The described work was the first to present evidence that plant miRNAs may pass the GI tract, enter the circulation and, most importantly, function in a cross-kingdom manner.

Zhang et al.’s report [89] motivated many scientists to investigate this issue in more depth. Some scientists successfully showed that plant, food-derived miRNAs may transfer to animals and regulate gene expression in their cells. In turn, others presented contradicting evidence of plant miRNA uptake and their influence on biological processes in animals. In this review, we summarize the current knowledge concerning cross-kingdom regulation by plant-derived miRNAs and consider how these molecules may transport from food to animal target cells. Finally, we briefly discuss how these findings may impact medicine and nutrition.

## 2. Detection and Function of Plant miRNAs in Animals—Supporting Evidence

Following Zhang’s article, research performed by Wang et al. detected a high number of small RNAs originating from various exogenous species, including dietary plants, in human plasma [90]. The most abundant miRNAs in the presented profile originated from *Zea mays* (corn) and *Oryza sativa* (rice). In addition to these cereal grains, Wang et al. were able to identify RNA molecules from other common food plant species, such as *Solanum lycopersicum* (tomato), *Glycine max* (soybean) and *Vitis vinifera* (grapes). Consistent with Zhang’s et al. study, small RNAs detected in human plasma were resistant to RNase A activity. Thus, the authors suggested that the investigated small RNA (sRNA) molecules may be accompanied by certain proteins, lipids and other particles that protect them from degradation [90].

In 2014, Liang et al. published the effects of a mouse feeding study in which animals received total RNA extract from *Brassica oleracea* (cabbage) [91]. Their findings showed that the investigated cabbage miR172 persisted through the GI tract for 72 h after feeding. Moreover, the authors were able to detect the mentioned miRNA within 2 h after feeding in mouse blood and different organs, such as the liver, spleen and kidney [91]. The next year, Hongwei Liang and coauthors published their results of an experiment in which volunteers drank watermelon juice or ate fruit (watermelon, banana, apple, orange, grape, mango and cantaloupe) [92]. Using quantitative reverse transcriptase PCR (qRT-PCR) and Northern blot methods, they were able to identify 10 selected plant miRNAs in human plasma at high basal levels [92].

The abundance of plant miRNAs in biological fluids was investigated by our team as well. Based on the evidence that endogenous miRNAs are present in breast milk and may regulate specific human gene expression [27,29,52], we decided to evaluate whether potentially food-derived miRNA molecules can be found in the breast milk from mammalian species [93]. The restrictive bioinformatics analysis of publicly available, raw data from high-throughput sequencing studies on miRNA composition in human and porcine breast milk exosomes led to identification of several plant miRNA species, e.g., MIR168a, MIR156a, MIR166a, MIR319b and MIR167d. Most of the identified plant molecules belong to evolutionarily conserved MIR families [93]. It is also important to note that the revealed plant miRNA profiles from mammalian breast milk were similar to the composition in human blood presented by Zhang et al. [89].

Two separate studies carried out by Yang et al. [94,95] demonstrated that diet-derived small RNAs are present in the sera and urine of plant-consuming animals. Their studies were focused on MIR2911 from the *Lonicera japonica* (honeysuckle) herb. The MIR2911 molecule was shown to appear in the circulation 3 days after starting the diet, and its level was associated with dietary intake levels. After the end of feeding, miRNA remained in the examined fluids for 48 h [94]. Yang et al. showed additionally that dietary MIR2911 in sera was not associated with the AGO2 protein and its uptake was not related to perturbations in gut permeability or the microbiome [95]. However, they proposed that certain diet or GI injuries may facilitate the delivery of diet-derived sRNAs [94].

The previously discussed MIR2911 molecule has also become a topic of interest of Zhang’s group, who published the initial study on cross-kingdom gene expression regulation by plant MIR168a [89]. In 2014, mentioned authors reported that drinking or eating a boiled honeysuckle decoction led to the elevation of the MIR2911 level in the sera and lungs of mice [96]. Additionally, the luciferase reporter assay and in vivo experiments showed that MIR2911 is able to target influenza A virus (IAV), particularly H1N1, H5N1, and H7N9, and consequently inhibit its replication and reduce mouse mortality. Intriguingly, honeysuckle has been known in traditional Chinese medicine for centuries and is used to treat influenza infections. Another interesting observation that can be drawn from the described study is that boiling of honeysuckle did not lead to the degradation of MIR2911 [96]. This is consistent with other research, which demonstrated that storage, processing and cooking do not abolish plant miRNA in food sources and that plant-derived miRNAs can survive in a simulated digestion system for at least 75 min [97]. The same authors of the above-described MIR2911 study went a step further and recently published their results showing that plant food-derived sRNAs are present in human umbilical blood and amniotic fluids [98]. Therefore, they proposed that miRNAs can pass through the placenta and enter the fetus. Because most of the investigated MIR2911 molecules were present in MVs after feeding, the transplacental sRNA transport was suggested to be mediated by these carriers [98]. To further explore the properties of small RNAs that passed through the placenta, Li et al. performed experiments revealing that after feeding, the synthetic small interfering RNA (siRNA) level was significantly elevated in the mouse fetus, while the siRNA target alpha-fetoprotein (AFP) level was downregulated [98].

The therapeutic potential of plant-based miRNAs was also recently confirmed by two different groups. Using a colon cancer mouse model, one study reported that the oral administration of a cocktail of three tumor suppressor miRNAs with methyl groups at the 2′ position of the 3′ nucleotide (mimicking plant miRNAs) reduced the colon tumor burden [99]. The second study found that plant miR159 is present in a woman’s sera and its level is inversely correlated with breast cancer morbidity and progression [100]. Most of the identified miR159 was abundant in extracellular vesicles (EV). More importantly, in vitro experiments performed by the mentioned group revealed that synthetic miR159 is capable of reducing the proliferation of breast cancer cells by targeting sequence of the 3′ untranslated region (UTR) of Transcription Factor 7 (TCF7) mRNA. In turn, the oral administration of miR159 mimic significantly inhibited the growth of xerograph breast tumors in mice [100].

## 3. Detection and Function of Plant miRNAs in Animals—Contradicting Evidence

Along with all of the presented research supporting the dietary uptake and cross-kingdom function of plant miRNAs, a large amount of contradicting evidence was also published, including criticisms of Zhang et al.’s study [101,102]. The possibility of batch effects, contamination or technical variability resulting from RNA extraction, library preparation and sequencing was discussed. Moreover, the strategy of pooling individual samples was also described. It has been suggested that a minority of donors could be responsible for most of the plant pooled reads. In turn, the quantity of raw rice given to mice in feeding experiments was high beyond the normal food intake level [101]. The stoichiometric and delivering considerations for plant–mammal sRNA communication were critically discussed in a book chapter written by Kenneth W. Witwer entitled “Non-coding RNAs and Inter-kingdom Communication” [103].

Correspondingly, several independent research groups have reported negative results of their plant-based miRNAs feeding studies in a variety of insects and animals. Snow et al. found a substantial level of plant MIR156a, MIR159a and MIR169a in a diet commonly consumed by humans, mice and honeybees [104]. However, in the plasma of healthy athletes who routinely eat fruits (e.g., bananas, apples and avocados), listed miRNAs could not be detected. Similar negative findings were shown for honeybees, which received pollen, honey and nectar as a food source, and in mice on a vegetarian/soy or avocado diet [104]. To expand the honeybee feeding study, Snow et al. performed and published further research in which they elucidated the level of plant miRNAs in different tissues of newly eclosed, nurse and forager bees. Their results showed that honeybees are able to ingest high levels of pollen-derived miRNAs; however, the systemic levels of these molecules are far below biologically relevant concentrations [105].

An additional study performed by Witwer et al. also failed to detect plant-derived miRNAs in animal plasma [106]. In their research, the authors measured plant uptake in two pigtail macaques fed a soy- and fruit-based mixture. The levels of certain plant miRNAs in the blood were evaluated before and after (1, 4 and 12 h) ingestion by qRT-PCR and droplet digital PCR. Although Witwer et al. observed very low levels of some of the investigated molecules, these low levels were interpreted as a result of non-specific amplification [106].

Recently, another research group aimed to detect plant miRNAs in the plasma of healthy volunteers that usually consume extra virgin olive oil (EVOO) [107]. They evaluated the abundance of plant miRNAs 2 h after the ingestion of EVOO using the high-throughput sequencing approach. Similarly to the studies discussed above, the authors failed to identify plant miRNAs in the mentioned body fluids. Micó et al. assumed that miRNAs present in plant-derived products, such as EVOO or beer, could be absorbed by the human gut and enter the circulation. However, primarily, they did not detect substantial quantities of plant miRNAs in the EVOO and beer samples [107].

The initial study by Zhang et al. [89] sparked also discussion on genetically modified organisms (GMOs). Many web services and magazines started to publish articles and comments concerning the unintended effect of miRNAs derived from consumed GMOs [108,109,110,111,112]. Despite the fact that the study by Zhang et al. did not directly address GMOs, the situation became so “uncomfortable” that scientists from the Monsanto corporation, the leading producer of genetically engineered seeds, published (on-line) their technical analysis of the Zhang et al. study [113] and a review discussing the safety of food from biotechnology-derived crops [114]. Moreover, together with researchers from the miRagen company, they made an effort to replicate the initial Zhang et al. experiment [115]. In their study, mice received one of three dietary formulations: standard chow, a nutritionally sufficient diet containing 41% rice or raw rice. Unfortunately, no or very little plant miRNA was detected in the plasma and organs of animals fed any of these diets. In turn, the levels of LDL in the mouse liver were increased, but the expression of the investigated LDLRAP1 protein remained unchanged across all examined groups [115]. Researchers from the Monsanto corporation performed additionally a survey of a large number of publically available sRNA datasets from animal fluids and tissues [116]. Their bioinformatics analyses revealed that of the 83 RNA datasets examined, 63 had at least one sequence representing plant miRNA. The most abundant molecule was MIR168, with a sequence typical of monocot plant species. The mentioned miRNA was also found in datasets for insects that did not feed on monocot plants, complemented by sequencing data from the scientist’s own insect feeding experiment. Based on the mentioned survey, the researchers from the Monsanto corporation assumed that the plant miRNAs observed in animal datasets may originate in the process of sequencing [116].

## 4. miRNA Transport from Cell to Cell 

Beyond competing arguments on whether the cross-kingdom regulation of gene expression by plant miRNAs (abundant even in low amounts) is possible, questions concerning how these molecules can pass through the GI tract, enter the circulation and transport from cell to cell have also been raised. In recent years, several intracellular carriers of endogenously originating miRNAs have been identified, including microvesicle (MV) compartments, which are membrane-derived vesicles released from various types of cells [117,118,119,120]. Based on their origin, size and mechanism of formulation, they can be divided into: (1) shedding vesicles (SVs), which directly bud from the cell surface; (2) exosomes, which are derived from the endosomal membrane; and (3) apoptotic bodies, which are released in response to apoptotic stimuli [121,122,123]. Listed vesicles were shown to protect miRNAs from degradation by RNases [117,124,125,126]. In some plants, exosome-like nanoparticles (called EPDENs) have been identified and were shown to carry proteins, lipids, and miRNAs [76,77,127,128]. One of the published studies suggested that EPDENs may mediate interspecies communication and induce the expression of certain human genes [128]. Extracellular miRNAs can be alternatively transported by lipoproteins, namely high-density lipoproteins (HDL) and low-density lipoproteins (LDL) [129,130]. As the main functional component of the microRNA ribonucleoprotein complex (miRNP), the AGO protein has also been observed to carry miRNA molecules [37,131].

Another issue is related to the passage of plant miRNAs through the GI tract. One option is that the intestinal epithelial cells take up miRNAs from food. In *Caenorhabditis elegans*, a ubiquitously expressed transmembrane systemic RNA interference defective protein 1 (SID-1) was shown to mediate the passive cellular uptake of double stranded RNAs (dsRNAs) [132,133]. In turn, the SID-2 protein localized in the intestine luminal membrane was thought to mediate the endocytosis of dsRNAs from the lumen [134]. Two homologous proteins of SID-1, SID1 transmembrane family member 1 (SIDT1) and SID1 transmembrane family member 2 (SIDT2), were also identified in most vertebrates. Their exact biochemical properties are still under investigation. However, previous reports have suggested that mentioned proteins may have similar functions to those present in *C. elegans*. Namely, human SIDT1 in pancreatic cells was shown to enhance siRNA uptake while in embryonic kidney cells, to mediate the intracellular transport of small RNAs [135,136,137]. Although many gaps in the body of knowledge on miRNA transport need to be filled, it can be assumed that the miRNA pathway from plant food sources to recipient cells may be as follows: by consuming plant material, we crush it mechanistically by oral activity and partially digest it by various enzymes in our mouth/stomach. During these processes, plant miRNAs are released from destroyed cells and transferred to the small intestine. After being incorporated into certain proteins or vesicles, they are secreted and transported to the target cells via the circulatory system.

## 5. Conclusions 

Despite their small size, miRNA molecules possess huge potential due to their ability to regulate target gene expression. miRNA profiles have been shown to correlate with many human diseases and pathological stages, which makes them even more attractive for scientists and the pharmaceutical industry. The ability of miRNAs to regulate gene expression in a cross-kingdom manner was already shown for viruses and parasites, whereas Zhang et al. were the first to demonstrate these intriguing properties for plant molecules [89]. Their study launched a wave of publications competing in arguments and started fierce debate among scientists that continues to this day. Table 1 summarizes all the arguments presented in this review. The results of Zhang et al. [89] came also as an attractive hypothesis that may reveal the molecular mechanism of certain folk remedies. The plant kingdom is full of species that have been used for their medical properties since the dawn of history. So far, some of these properties are associated with minerals, vitamins, antioxidants, proteins or other small molecules, whereas mechanisms regarding rest of them remain unknown. Therefore, in light of the presented studies, miRNAs may be another component responsible for the medical properties of plants. Considering this in a boarder sense, the well-known Hippocrates quote “Let food be thy medicine and medicine be thy food” may gain a new meaning. Without a doubt, the cross-kingdom transfer of plant miRNAs and their therapeutic potential need to be further investigated and unambiguously confirmed. Any potential risk should also be seriously considered. This process will facilitate the development of new approaches to prevent or treat several known human diseases.

## Figures and Tables

**Table 1:** Summary of arguments for and against the theory of cross-kingdom communication by plant microRNAs (miRNAs) presented by recent studies.

Evidence	Results	Methods Used	References
**Supporting Evidence**	MicroRNA MIR168 molecules from *Oryza sativa* (rice) were abundant in human serum. MIR168 were present in microvesicles (MV) and could associate with the human Argonaute 2 (AGO2). MIR168 negatively regulated the expression of the human low-density lipoprotein receptor adaptor protein 1 (LDLRAP1) protein in liver, which in turn resulted in low-density lipoprotein (LDL) increase in plasma.	High-throughput sequencing, Quantitative reverse transcriptase PCR (qRT-PCR) and semi-quantitative RT-PCR, Northern blot, Bioinformatics analysis, Luciferase reporter assay, Western blot, AGO2 immunoprecipitation.	[89]
	Certain plant miRNAs were present in human plasma and may be associated with some particles protecting them from degradation.	High-throughput sequencing	[90]
	MIR172 from *Brassica oleracea* (cabbage) was present in mouse serum and various tissues after animal feeding with plant extract.	Electrophoresis, RT-PCR.	[91]
	Ten selected plant miRNAs were present in the plasma of volunteers drinking watermelon juice or eating different fruits. Plant miRNAs were largely encapsulated in MV.	qRT-PCR, Northern blot.	[92]
	Several plant miRNAs were identified in exosomes isolated from mammalian breast milk.	Bioinformatics analysis	[93]
	MIR2911 and MIR168 from *Lonicera japonica* (honeysuckle) were found in the sera and urine of mice fed a plant-based diet. The level of a molecule was associated with the dietary intake level. Specific diet or/and alterations in intestinal permeability may improve plant miRNA absorption.	qRT-PCR and digital droplet PCR, AGO2 immunoprecipitation, Fluorometry.	[94,95]
	MIR2911 from *Lonicera japonica* (honeysuckle) was found in the sera and lungs of mice fed a honeysuckle decoction. MIR2911 directly targeted Influenza A virus and inhibited its replication.	Luciferase reporter assay, Bioinformatics analysis, High-throughput sequencing, qRT-PCR, Northern blot, Ago2 immunoprecipitation, Western blot.	[96]
	Plant miRNAs were stable in a simulated human digestion system for over 1 h. Plant miRNAs could survive storing, cooking and other types of processing.	Drug dissolution tester, qRT-PCR.	[97]
	Plant miRNA was found in human umbilical cord blood and amniotic fluids. MIR2911 from *Lonicera japonica* (honeysuckle) could transfer through the placenta and enter fetal liver in mice. MIR2911 was primarily present in MV.	qRT-PCR, High-throughput sequencing, Confocal microscopy analysis.	[98]
	Cocktail of 3 miRNA mimicking small RNAs produced in plants reduced colon cancer.	Periodate oxidation and qRT-PCR, Dissecting microscope.	[99]
	Plant MIR159 was present in a woman’s sera. Oral administration of synthetic MIR159 suppressed the growth of breast tumors in mice. MIR159 was predominantly present in extracellular vesicles (EV).	Sodium periodate qPCR and qRT-PCR, High-throughput sequencing, Bioinformatics analysis, RISCTRAP assay, Dual-luciferase reporter assay, In situ hybridization, Immunohistochemistry, Western blot.	[100]
**Contradicting Evidence**	Critical discussions on plant-mammal sRNA communication (including the study by Zhang et al.)—issues: Contamination, Batch effects, Technical variability, Stability of plant miRNAs in mammalian organisms, Transport of plant miRNAs to mammalian cells, Functionality of plant miRNAs, Stoichiometry, Safety of biotech-derived food.	Not experimental—discussion based on current knowledge/previous studies, Bioinformatics re-analysis of the study by Zhang et al.	[101,102,103,113,114]
	Plant miRNAs could not be detected in the plasma of healthy athletes consuming fruits. Plant miRNAs were undetectable in the plasma and tissues of mice on a vegetarian diet. Plant miRNAs were undetectable in tissues of newly eclosed, nurse and forager honeybees fed with pollen/honey/nectar, or their systemic level was below relevant, active concentrations.	qRT-PCR	[104,105]
	Plant miRNAs were not present in blood obtained before and after feeding of pigtailed macaques with a miRNA-rich plant-based substance.	qRT-PCR and droplet digital PCR	[106]
	Plant miRNAs could not be detected in the plasma of volunteers consuming extra virgin olive oil.	High-throughput sequencing	[107]
	None or very few plant miRNAs were found in the liver and plasma of mice fed any of the described diet regimens. The LDL level in mouse plasma was increased; however, this was proposed as a result of the short-term nutritional impact of the rice diet following the fasting period. The LDLRAP1 protein level was not reduced in the mouse liver.	High-throughput sequencing, qPCR, Enzyme-linked immunosorbent assay (ELISA).	[115]
	Plant miRNAs were observed in public animal sRNA datasets and sequencing data from insect feeding experiments. Identified plant miRNAs may be an artifact of the sequencing methodology.	High-throughput sequencing, Bioinformatics analysis, Northern blot.	[116]

## References

[1] Jonas S., Izaurralde E. (2015). Towards a molecular understanding of microRNA-mediated gene silencing. Nat. Rev. Genet..

[2] Ipsaro J.J., Joshua-Tor L. (2015). From guide to target: Molecular insights into eukaryotic RNA-interference machinery. Nat. Struct. Mol. Biol..

[3] Huntzinger E., Izaurralde E. (2011). Gene silencing by microRNAs: Contributions of translational repression and mRNA decay. Nat. Rev. Genet..

[4] Fabian M.R., Sonenberg N. (2012). The mechanics of miRNA-mediated gene silencing: A look under the hood of mirisc. Nat. Struct. Mol. Biol..

[5] Friedman R.C., Farh K.K., Burge C.B., Bartel D.P. (2009). Most mammalian mRNAs are conserved targets of microRNAs. Genome Res..

[6] Zhang B., Wang Q., Pan X. (2007). MicroRNAs and their regulatory roles in animals and plants. J. Cell. Physiol..

[7] Alvarez-Garcia I., Miska E.A. (2005). MicroRNA functions in animal development and human disease. Development.

[8] Miska E.A. (2005). How microRNAs control cell division, differentiation and death. Curr. Opin. Genet. Dev..

[9] Bushati N., Cohen S.M. (2007). MicroRNA functions. Annu. Rev. Cell Dev. Biol..

[10] Dugas D.V., Bartel B. (2004). MicroRNA regulation of gene expression in plants. Curr. Opin. Plant Biol..

[11] Kruszka K., Pieczynski M., Windels D., Bielewicz D., Jarmolowski A., Szweykowska-Kulinska Z., Vazquez F. (2012). Role of microRNAs and other sRNAs of plants in their changing environments. J. Plant Physiol..

[12] Shomron N., Golan D., Hornstein E. (2009). An evolutionary perspective of animal microRNAs and their targets. J. Biomed. Biotechnol..

[13] Jones-Rhoades M.W. (2012). Conservation and divergence in plant microRNAs. Plant Mol. Biol..

[14] Molnar A., Schwach F., Studholme D.J., Thuenemann E.C., Baulcombe D.C. (2007). MiRNAs control gene expression in the single-cell alga chlamydomonas reinhardtii. Nature.

[15] Grundhoff A., Sullivan C.S. (2011). Virus-encoded microRNAs. Virology.

[16] Kang K., Zhong J., Jiang L., Liu G., Gou C.Y., Wu Q., Wang Y., Luo J., Gou D. (2013). Identification of microRNA-like RNAs in the filamentous fungus trichoderma reesei by solexa sequencing. PLoS ONE.

[17] Zhou J., Fu Y., Xie J., Li B., Jiang D., Li G., Cheng J. (2012). Identification of microRNA-like RNAs in a plant pathogenic fungus sclerotinia sclerotiorum by high-throughput sequencing. Mol. Genet. Genom..

[18] Kozomara A., Griffiths-Jones S. (2014). Mirbase: Annotating high confidence microRNAs using deep sequencing data. Nucleic Acids Res..

[19] Cortez M.A., Calin G.A. (2009). MicroRNA identification in plasma and serum: A new tool to diagnose and monitor diseases. Expert Opin. Biol. Ther..

[20] Song M.Y., Pan K.F., Su H.J., Zhang L., Ma J.L., Li J.Y., Yuasa Y., Kang D., Kim Y.S., You W.C. (2012). Identification of serum microRNAs as novel non-invasive biomarkers for early detection of gastric cancer. PLoS ONE.

[21] Chen X., Ba Y., Ma L., Cai X., Yin Y., Wang K., Guo J., Zhang Y., Chen J., Guo X. (2008). Characterization of microRNAs in serum: A novel class of biomarkers for diagnosis of cancer and other diseases. Cell Res..

[22] Hunter M.P., Ismail N., Zhang X., Aguda B.D., Lee E.J., Yu L., Xiao T., Schafer J., Lee M.L., Schmittgen T.D. (2008). Detection of microRNA expression in human peripheral blood microvesicles. PLoS ONE.

[23] Van der Eerden B.C., Alves R.D., Kockx C.E., Ozgur Z., Schreuders-Koedam M., van de Peppel J., van Ijcken W.F., van Leeuwen J.P. (2015). Identification of microRNAs in human plasma. Methods Mol. Biol..

[24] Pavkovic M., Riefke B., Ellinger-Ziegelbauer H. (2014). Urinary microRNA profiling for identification of biomarkers after cisplatin-induced kidney injury. Toxicology.

[25] Korzeniewski N., Tosev G., Pahernik S., Hadaschik B., Hohenfellner M., Duensing S. (2015). Identification of cell-free microRNAs in the urine of patients with prostate cancer. Urol. Oncol..

[26] Mengual L., Lozano J.J., Ingelmo-Torres M., Gazquez C., Ribal M.J., Alcaraz A. (2013). Using microRNA profiling in urine samples to develop a non-invasive test for bladder cancer. Int. J. Cancer.

[27] Zhou Q., Li M., Wang X., Li Q., Wang T., Zhu Q., Zhou X., Wang X., Gao X., Li X. (2012). Immune-related microRNAs are abundant in breast milk exosomes. Int. J. Biol. Sci..

[28] Alsaweed M., Lai C.T., Hartmann P.E., Geddes D.T., Kakulas F. (2016). Human milk cells contain numerous miRNAs that may change with milk removal and regulate multiple physiological processes. Int. J. Mol. Sci..

[29] Kosaka N., Izumi H., Sekine K., Ochiya T. (2010). MicroRNA as a new immune-regulatory agent in breast milk. Silence.

[30] Ogawa Y., Taketomi Y., Murakami M., Tsujimoto M., Yanoshita R. (2013). Small RNA transcriptomes of two types of exosomes in human whole saliva determined by next generation sequencing. Biol. Pharm. Bull..

[31] Courts C., Madea B. (2011). Specific micro-RNA signatures for the detection of saliva and blood in forensic body-fluid identification. J. Forensic Sci..

[32] Weber J.A., Baxter D.H., Zhang S., Huang D.Y., Huang K.H., Lee M.J., Galas D.J., Wang K. (2010). The microRNA spectrum in 12 body fluids. Clin. Chem..

[33] Liu T., Cheng W., Gao Y., Wang H., Liu Z. (2012). Microarray analysis of microRNA expression patterns in the semen of infertile men with semen abnormalities. Mol. Med. Rep..

[34] Wu W., Hu Z., Qin Y., Dong J., Dai J., Lu C., Zhang W., Shen H., Xia Y., Wang X. (2012). Seminal plasma microRNAs: Potential biomarkers for spermatogenesis status. Mol. Hum. Reprod..

[35] Burgos K.L., Javaherian A., Bomprezzi R., Ghaffari L., Rhodes S., Courtright A., Tembe W., Kim S., Metpally R., van Keuren-Jensen K. (2013). Identification of extracellular miRNA in human cerebrospinal fluid by next-generation sequencing. RNA.

[36] Ge Q., Zhou Y., Lu J., Bai Y., Xie X., Lu Z. (2014). MiRNA in plasma exosome is stable under different storage conditions. Molecules.

[37] Turchinovich A., Weiz L., Langheinz A., Burwinkel B. (2011). Characterization of extracellular circulating microRNA. Nucleic Acids Res..

[38] Li Y., Jiang Z., Xu L., Yao H., Guo J., Ding X. (2011). Stability analysis of liver cancer-related microRNAs. Acta Biochim. Biophys. Sin..

[39] Li H., Fan J., Yin Z., Wang F., Chen C., Wang D.W. (2016). Identification of cardiac-related circulating microRNA profile in human chronic heart failure. Oncotarget.

[40] Duy J., Koehler J.W., Honko A.N., Schoepp R.J., Wauquier N., Gonzalez J.P., Pitt M.L., Mucker E.M., Johnson J.C., O’Hearn A. (2016). Circulating microRNA profiles of ebola virus infection. Sci. Rep..

[41] Caserta S., Kern F., Cohen J., Drage S., Newbury S.F., Llewelyn M.J. (2016). Circulating plasma microRNAs can differentiate human sepsis and systemic inflammatory response syndrome (sirs). Sci. Rep..

[42] Pescador N., Perez-Barba M., Ibarra J.M., Corbaton A., Martinez-Larrad M.T., Serrano-Rios M. (2013). Serum circulating microRNA profiling for identification of potential type 2 diabetes and obesity biomarkers. PLoS ONE.

[43] Zhang C., Wang C., Chen X., Yang C., Li K., Wang J., Dai J., Hu Z., Zhou X., Chen L. (2010). Expression profile of microRNAs in serum: A fingerprint for esophageal squamous cell carcinoma. Clin. Chem..

[44] Tsujiura M., Ichikawa D., Komatsu S., Shiozaki A., Takeshita H., Kosuga T., Konishi H., Morimura R., Deguchi K., Fujiwara H. (2010). Circulating microRNAs in plasma of patients with gastric cancers. Br. J. Cancer.

[45] De Guire V., Robitaille R., Tetreault N., Guerin R., Menard C., Bambace N., Sapieha P. (2013). Circulating miRNAs as sensitive and specific biomarkers for the diagnosis and monitoring of human diseases: Promises and challenges. Clin. Biochem..

[46] Cortez M.A., Bueso-Ramos C., Ferdin J., Lopez-Berestein G., Sood A.K., Calin G.A. (2011). MicroRNAs in body fluids--the mix of hormones and biomarkers. Nat. Rev. Clin. Oncol..

[47] Jacob N.K., Cooley J.V., Yee T.N., Jacob J., Alder H., Wickramasinghe P., Maclean K.H., Chakravarti A. (2013). Identification of sensitive serum microRNA biomarkers for radiation biodosimetry. PLoS ONE.

[48] Silva S.S., Lopes C., Teixeira A.L., Carneiro de Sousa M.J., Medeiros R. (2015). Forensic miRNA: Potential biomarker for body fluids?. Forensic Sci. Int. Genet..

[49] Parigi S.M., Eldh M., Larssen P., Gabrielsson S., Villablanca E.J. (2015). Breast milk and solid food shaping intestinal immunity. Front. Immunol..

[50] Calder P.C., Krauss-Etschmann S., de Jong E.C., Dupont C., Frick J.S., Frokiaer H., Heinrich J., Garn H., Koletzko S., Lack G. (2006). Early nutrition and immunity—Progress and perspectives. Br. J. Nutr..

[51] Rogier E.W., Frantz A.L., Bruno M.E., Wedlund L., Cohen D.A., Stromberg A.J., Kaetzel C.S. (2014). Secretory antibodies in breast milk promote long-term intestinal homeostasis by regulating the gut microbiota and host gene expression. Proc. Natl. Acad. Sci. USA.

[52] Gu Y., Li M., Wang T., Liang Y., Zhong Z., Wang X., Zhou Q., Chen L., Lang Q., He Z. (2012). Lactation-related microRNA expression profiles of porcine breast milk exosomes. PLoS ONE.

[53] Chen T., Xi Q.Y., Ye R.S., Cheng X., Qi Q.E., Wang S.B., Shu G., Wang L.N., Zhu X.T., Jiang Q.Y. (2014). Exploration of microRNAs in porcine milk exosomes. BMC Genom..

[54] Izumi H., Kosaka N., Shimizu T., Sekine K., Ochiya T., Takase M. (2012). Bovine milk contains microRNA and messenger RNA that are stable under degradative conditions. J. Dairy Sci..

[55] Oh S., Park M.R., Son S.J., Kim Y. (2015). Comparison of total RNA isolation methods for analysis of immune-related microRNAs in market milks. Korean J. Food Sci. Anim. Resour..

[56] Chen X., Gao C., Li H., Huang L., Sun Q., Dong Y., Tian C., Gao S., Dong H., Guan D. (2010). Identification and characterization of microRNAs in raw milk during different periods of lactation, commercial fluid, and powdered milk products. Cell Res..

[57] Sun J., Aswath K., Schroeder S.G., Lippolis J.D., Reinhardt T.A., Sonstegard T.S. (2015). MicroRNA expression profiles of bovine milk exosomes in response to staphylococcus aureus infection. BMC Genom..

[58] Izumi H., Tsuda M., Sato Y., Kosaka N., Ochiya T., Iwamoto H., Namba K., Takeda Y. (2015). Bovine milk exosomes contain microRNA and mRNA and are taken up by human macrophages. J. Dairy Sci..

[59] Sun Q., Chen X., Yu J., Zen K., Zhang C.Y., Li L. (2013). Immune modulatory function of abundant immune-related microRNAs in microvesicles from bovine colostrum. Protein Cell.

[60] Benmoussa A., Lee C.H., Laffont B., Savard P., Laugier J., Boilard E., Gilbert C., Fliss I., Provost P. (2016). Commercial dairy cow milk microRNAs resist digestion under simulated gastrointestinal tract conditions. J. Nutr..

[61] Howard K.M., Jati Kusuma R., Baier S.R., Friemel T., Markham L., Vanamala J., Zempleni J. (2015). Loss of miRNAs during processing and storage of cow’s (bos taurus) milk. J. Agric. Food Chem..

[62] Baier S.R., Nguyen C., Xie F., Wood J.R., Zempleni J. (2014). MicroRNAs are absorbed in biologically meaningful amounts from nutritionally relevant doses of cow milk and affect gene expression in peripheral blood mononuclear cells, hek-293 kidney cell cultures, and mouse livers. J. Nutr..

[63] Auerbach A., Vyas G., Li A., Halushka M., Witwer K. (2016). Uptake of dietary milk miRNAs by adult humans: A validation study. F1000Reserch.

[64] Witwer K.W. (2014). Diet-responsive mammalian miRNAs are likely endogenous. J. Nutr..

[65] Lee R.C., Feinbaum R.L., Ambros V. (1993). The *C. elegans* heterochronic gene lin-4 encodes small RNAs with antisense complementarity to lin-14. Cell.

[66] Wightman B., Ha I., Ruvkun G. (1993). Posttranscriptional regulation of the heterochronic gene lin-14 by lin-4 mediates temporal pattern formation in *C. elegans*. Cell.

[67] Van Rooij E., Kauppinen S. (2014). Development of microRNA therapeutics is coming of age. EMBO Mol. Med..

[68] Lam J.K., Chow M.Y., Zhang Y., Leung S.W. (2015). SiRNA versus miRNA as therapeutics for gene silencing. Mol. Ther. Nucleic Acids.

[69] Schmidt M.F. (2014). Drug target miRNAs: Chances and challenges. Trends Biotechnol..

[70] Zhang Y., Wang Z., Gemeinhart R.A. (2013). Progress in microRNA delivery. J. Control. Release.

[71] Deleavey G.F., Damha M.J. (2012). Designing chemically modified oligonucleotides for targeted gene silencing. Chem. Biol..

[72] Cheng K., Mahato R.I. (2013). Advanced Delivery and Therapeutic Applications of Rnai.

[73] Xue H.Y., Guo P., Wen W.C., Wong H.L. (2015). Lipid-based nanocarriers for RNA delivery. Curr. Pharm. Des..

[74] Ohno S., Takanashi M., Sudo K., Ueda S., Ishikawa A., Matsuyama N., Fujita K., Mizutani T., Ohgi T., Ochiya T. (2013). Systemically injected exosomes targeted to EGFR deliver antitumor microRNA to breast cancer cells. Mol. Ther..

[75] Munagala R., Aqil F., Jeyabalan J., Gupta R.C. (2016). Bovine milk-derived exosomes for drug delivery. Cancer Lett..

[76] Wang B., Zhuang X., Deng Z.B., Jiang H., Mu J., Wang Q., Xiang X., Guo H., Zhang L., Dryden G. (2014). Targeted drug delivery to intestinal macrophages by bioactive nanovesicles released from grapefruit. Mol. Ther..

[77] Ju S., Mu J., Dokland T., Zhuang X., Wang Q., Jiang H., Xiang X., Deng Z.B., Wang B., Zhang L. (2013). Grape exosome-like nanoparticles induce intestinal stem cells and protect mice from dss-induced colitis. Mol. Ther..

[78] Smyth T., Kullberg M., Malik N., Smith-Jones P., Graner M.W., Anchordoquy T.J. (2015). Biodistribution and delivery efficiency of unmodified tumor-derived exosomes. J. Control. Release.

[79] Kooijmans S.A., Vader P., van Dommelen S.M., van Solinge W.W., Schiffelers R.M. (2012). Exosome mimetics: A novel class of drug delivery systems. Int. J. Nanomed..

[80] Jang S.C., Kim O.Y., Yoon C.M., Choi D.S., Roh T.Y., Park J., Nilsson J., Lotvall J., Kim Y.K., Gho Y.S. (2013). Bioinspired exosome-mimetic nanovesicles for targeted delivery of chemotherapeutics to malignant tumors. ACS Nano.

[81] Alvarez-Erviti L., Seow Y., Yin H., Betts C., Lakhal S., Wood M.J. (2011). Delivery of siRNA to the mouse brain by systemic injection of targeted exosomes. Nat. Biotechnol..

[82] Tian Y., Li S., Song J., Ji T., Zhu M., Anderson G.J., Wei J., Nie G. (2014). A doxorubicin delivery platform using engineered natural membrane vesicle exosomes for targeted tumor therapy. Biomaterials.

[83] Rana S., Yue S., Stadel D., Zoller M. (2012). Toward tailored exosomes: The exosomal tetraspanin web contributes to target cell selection. Int. J. Biochem. Cell Biol..

[84] Hung M.E., Leonard J.N. (2015). Stabilization of exosome-targeting peptides via engineered glycosylation. J. Biol. Chem..

[85] Choy E.Y., Siu K.L., Kok K.H., Lung R.W., Tsang C.M., To K.F., Kwong D.L., Tsao S.W., Jin D.Y. (2008). An epstein-barr virus-encoded microRNA targets puma to promote host cell survival. J. Exp. Med..

[86] Lecellier C.H., Dunoyer P., Arar K., Lehmann-Che J., Eyquem S., Himber C., Saib A., Voinnet O. (2005). A cellular microRNA mediates antiviral defense in human cells. Science.

[87] Huang J., Wang F., Argyris E., Chen K., Liang Z., Tian H., Huang W., Squires K., Verlinghieri G., Zhang H. (2007). Cellular microRNAs contribute to HIV-1 latency in resting primary CD4+ T lymphocytes. Nat. Med..

[88] LaMonte G., Philip N., Reardon J., Lacsina J.R., Majoros W., Chapman L., Thornburg C.D., Telen M.J., Ohler U., Nicchitta C.V. (2012). Translocation of sickle cell erythrocyte microRNAs into plasmodium falciparum inhibits parasite translation and contributes to malaria resistance. Cell Host Microbe.

[89] Zhang L., Hou D., Chen X., Li D., Zhu L., Zhang Y., Li J., Bian Z., Liang X., Cai X. (2012). Exogenous plant mir168a specifically targets mammalian lDLRAP1: Evidence of cross-kingdom regulation by microRNA. Cell Res..

[90] Wang K., Li H., Yuan Y., Etheridge A., Zhou Y., Huang D., Wilmes P., Galas D. (2012). The complex exogenous RNA spectra in human plasma: An interface with human gut biota?. PLoS ONE.

[91] Liang G., Zhu Y., Sun B., Shao Y., Jing A., Wang J., Xiao Z. (2014). Assessing the survival of exogenous plant microRNA in mice. Food Sci. Nutr..

[92] Liang H., Zhang S., Fu Z., Wang Y., Wang N., Liu Y., Zhao C., Wu J., Hu Y., Zhang J. (2015). Effective detection and quantification of dietetically absorbed plant microRNAs in human plasma. J. Nutr. Biochem..

[93] Lukasik A., Zielenkiewicz P. (2014). In silico identification of plant miRNAs in mammalian breast milk exosomes—A small step forward?. PLoS ONE.

[94] Yang J., Farmer L.M., Agyekum A.A., Hirschi K.D. (2015). Detection of dietary plant-based small RNAs in animals. Cell Res..

[95] Yang J., Farmer L.M., Agyekum A.A., Elbaz-Younes I., Hirschi K.D. (2015). Detection of an abundant plant-based small RNA in healthy consumers. PLoS ONE.

[96] Zhou Z., Li X., Liu J., Dong L., Chen Q., Liu J., Kong H., Zhang Q., Qi X., Hou D. (2015). Honeysuckle-encoded atypical microRNA2911 directly targets influenza a viruses. Cell Res..

[97] Philip A., Ferro V.A., Tate R.J. (2015). Determination of the potential bioavailability of plant microRNAs using a simulated human digestion process. Mol. Nutr. Food Res..

[98] Li J., Zhang Y., Li D., Liu Y., Chu D., Jiang X., Hou D., Zen K., Zhang C.Y. (2015). Small non-coding RNAs transfer through mammalian placenta and directly regulate fetal gene expression. Protein Cell.

[99] Mlotshwa S., Pruss G.J., MacArthur J.L., Endres M.W., Davis C., Hofseth L.J., Pena M.M., Vance V. (2015). A novel chemopreventive strategy based on therapeutic microRNAs produced in plants. Cell Res..

[100] Chin A.R., Fong M.Y., Somlo G., Wu J., Swiderski P., Wu X., Wang S.E. (2016). Cross-kingdom inhibition of breast cancer growth by plant mir159. Cell Res..

[101] Witwer K.W., Hirschi K.D. (2014). Transfer and functional consequences of dietary microRNAs in vertebrates: Concepts in search of corroboration: Negative results challenge the hypothesis that dietary xenomirs cross the gut and regulate genes in ingesting vertebrates, but important questions persist. Bioessays.

[102] Witwer K.W. (2012). Xenomirs and miRNA homeostasis in health and disease: Evidence that diet and dietary miRNAs directly and indirectly influence circulating miRNA profiles. RNA Biol..

[103] Leitão A.L., Enguita F.J. (2016). Non-Coding Rnas and Inter-Kingdom Communication.

[104] Snow J.W., Hale A.E., Isaacs S.K., Baggish A.L., Chan S.Y. (2013). Ineffective delivery of diet-derived microRNAs to recipient animal organisms. RNA Biol..

[105] Masood M., Everett C.P., Chan S.Y., Snow J.W. (2016). Negligible uptake and transfer of diet-derived pollen microRNAs in adult honey bees. RNA Biol..

[106] Witwer K.W., McAlexander M.A., Queen S.E., Adams R.J. (2013). Real-time quantitative pcr and droplet digital pcr for plant miRNAs in mammalian blood provide little evidence for general uptake of dietary miRNAs: Limited evidence for general uptake of dietary plant xenomirs. RNA Biol..

[107] Mico V., Martin R., Lasuncion M.A., Ordovas J.M., Daimiel L. (2016). Unsuccessful detection of plant microRNAs in beer, extra virgin olive oil and human plasma after an acute ingestion of extra virgin olive oil. Plant Foods Hum. Nutr..

[108] Stevenson H. Gm Wheat May Damage Human Genetics Permanently. http://www.greenmedinfo.com/blog/gm-wheat-may-damage-human-genetics-permanently.

[109] Entine J. Metastisizing Misinformation about Gmos and Rna: Ugly Glare on Union of Concerned Scientists, Consumers Union. http://www.forbes.com/sites/jonentine/2013/11/12/metastisizing-misinformation-about-gmos-and-RNA-ugly-glare-on-union-of-concerned-scientists-consumers-union/#30f396e84203.

[110] Greenwood V. What you Eat Affects Your Genes: Rna from Rice Can Survive Digestion and Alter Gene Expression. http://blogs.discovermagazine.com/80beats/2011/09/21/what-you-eat-affects-your-genes-RNA-from-rice-can-survive-digestion-and-alter-gene-expression/#.V4DyafmLTIV.

[111] Levaux A. A Potential Danger of Genetic Modification. http://www.theatlantic.com/health/archive/2012/01/the-very-real-danger-of-genetically-modified-foods/251051/.

[112] Hodge A.-M.C. Food We Eat Might Control Our Genes. http://www.scientificamerican.com/article/vitamins-minerals-and-microrna/.

[113] Monsanto Technical Analysis: Zhang et al. http://gmopundit.blogspot.com/2012/01/monsanto-company-comments-on-much-hyped.html.

[114] Petrick J.S., Brower-Toland B., Jackson A.L., Kier L.D. (2013). Safety assessment of food and feed from biotechnology-derived crops employing RNA-mediated gene regulation to achieve desired traits: A scientific review. Regul. Toxicol. Pharmacol..

[115] Dickinson B., Zhang Y., Petrick J.S., Heck G., Ivashuta S., Marshall W.S. (2013). Lack of detectable oral bioavailability of plant microRNAs after feeding in mice. Nat. Biotechnol..

[116] Zhang Y., Wiggins B.E., Lawrence C., Petrick J., Ivashuta S., Heck G. (2012). Analysis of plant-derived miRNAs in animal small RNA datasets. BMC Genom..

[117] Valadi H., Ekstrom K., Bossios A., Sjostrand M., Lee J.J., Lotvall J.O. (2007). Exosome-mediated transfer of mRNAs and microRNAs is a novel mechanism of genetic exchange between cells. Nat. Cell Biol..

[118] Zernecke A., Bidzhekov K., Noels H., Shagdarsuren E., Gan L., Denecke B., Hristov M., Koppel T., Jahantigh M.N., Lutgens E. (2009). Delivery of microRNA-126 by apoptotic bodies induces cxcl12-dependent vascular protection. Sci. Signal..

[119] Zhang J., Li S., Li L., Li M., Guo C., Yao J., Mi S. (2015). Exosome and exosomal microRNA: Trafficking, sorting, and function. Genom. Proteom. Bioinform..

[120] Turchinovich A., Weiz L., Burwinkel B. (2012). Extracellular miRNAs: The mystery of their origin and function. Trends Biochem. Sci..

[121] Cocucci E., Racchetti G., Meldolesi J. (2009). Shedding microvesicles: Artefacts no more. Trends Cell Biol..

[122] Mathivanan S., Ji H., Simpson R.J. (2010). Exosomes: Extracellular organelles important in intercellular communication. J. Proteom..

[123] Van der Pol E., Boing A.N., Harrison P., Sturk A., Nieuwland R. (2012). Classification, functions, and clinical relevance of extracellular vesicles. Pharmacol. Rev..

[124] Huang X., Yuan T., Tschannen M., Sun Z., Jacob H., Du M., Liang M., Dittmar R.L., Liu Y., Liang M. (2013). Characterization of human plasma-derived exosomal RNAs by deep sequencing. BMC Genom..

[125] Keller S., Ridinger J., Rupp A.K., Janssen J.W., Altevogt P. (2011). Body fluid derived exosomes as a novel template for clinical diagnostics. J. Transl. Med..

[126] Cheng L., Sharples R.A., Scicluna B.J., Hill A.F. (2014). Exosomes provide a protective and enriched source of miRNA for biomarker profiling compared to intracellular and cell-free blood. J. Extracell. Vesicles.

[127] Yang J., Hotz T., Broadnax L., Yarmarkovich M., Elbaz-Younes I., Hirschi K.D. (2016). Anomalous uptake and circulatory characteristics of the plant-based small RNA mir2911. Sci. Rep..

[128] Mu J., Zhuang X., Wang Q., Jiang H., Deng Z.B., Wang B., Zhang L., Kakar S., Jun Y., Miller D. (2014). Interspecies communication between plant and mouse gut host cells through edible plant derived exosome-like nanoparticles. Mol. Nutr. Food Res..

[129] Vickers K.C., Palmisano B.T., Shoucri B.M., Shamburek R.D., Remaley A.T. (2011). MicroRNAs are transported in plasma and delivered to recipient cells by high-density lipoproteins. Nat. Cell Biol..

[130] Wagner J., Riwanto M., Besler C., Knau A., Fichtlscherer S., Roxe T., Zeiher A.M., Landmesser U., Dimmeler S. (2013). Characterization of levels and cellular transfer of circulating lipoprotein-bound microRNAs. Arterioscler. Thromb. Vasc. Biol..

[131] Arroyo J.D., Chevillet J.R., Kroh E.M., Ruf I.K., Pritchard C.C., Gibson D.F., Mitchell P.S., Bennett C.F., Pogosova-Agadjanyan E.L., Stirewalt D.L. (2011). Argonaute2 complexes carry a population of circulating microRNAs independent of vesicles in human plasma. Proc. Natl. Acad. Sci. USA.

[132] Feinberg E.H., Hunter C.P. (2003). Transport of dsRNA into cells by the transmembrane protein sid-1. Science.

[133] Shih J.D., Fitzgerald M.C., Sutherlin M., Hunter C.P. (2009). The sid-1 double-stranded RNA transporter is not selective for dsRNA length. RNA.

[134] McEwan D.L., Weisman A.S., Hunter C.P. (2012). Uptake of extracellular double-stranded RNA by sid-2. Mol. Cell.

[135] Duxbury M.S., Ashley S.W., Whang E.E. (2005). Rna interference: A mammalian sid-1 homologue enhances siRNA uptake and gene silencing efficacy in human cells. Biochem. Biophys. Res. Commun..

[136] Elhassan M.O., Christie J., Duxbury M.S. (2012). Homo sapiens systemic RNA interference-defective-1 transmembrane family member 1 (SIDT1) protein mediates contact-dependent small RNA transfer and microRNA-21-driven chemoresistance. J. Biol. Chem..

[137] Wolfrum C., Shi S., Jayaprakash K.N., Jayaraman M., Wang G., Pandey R.K., Rajeev K.G., Nakayama T., Charrise K., Ndungo E.M. (2007). Mechanisms and optimization of in vivo delivery of lipophilic siRNAs. Nat. Biotechnol..

